# Shape and Function of Interstitial Chemokine CCL21 Gradients Are Independent of Heparan Sulfates Produced by Lymphatic Endothelium

**DOI:** 10.3389/fimmu.2021.630002

**Published:** 2021-02-25

**Authors:** Kari Vaahtomeri, Christine Moussion, Robert Hauschild, Michael Sixt

**Affiliations:** ^1^ Institute of Science and Technology Austria (IST Austria), Klosterneuburg, Austria; ^2^ Wihuri Research Institute and Translational Cancer Medicine Research Program, University of Helsinki, Biomedicum Helsinki, Helsinki, Finland

**Keywords:** chemokine gradient, chemokine CCL21, chemotaxis, lymphatic system, lymphatic endothelium, dendritic cell, heparan sulfate

## Abstract

Gradients of chemokines and growth factors guide migrating cells and morphogenetic processes. Migration of antigen-presenting dendritic cells from the interstitium into the lymphatic system is dependent on chemokine CCL21, which is secreted by endothelial cells of the lymphatic capillary, binds heparan sulfates and forms gradients decaying into the interstitium. Despite the importance of CCL21 gradients, and chemokine gradients in general, the mechanisms of gradient formation are unclear. Studies on fibroblast growth factors have shown that limited diffusion is crucial for gradient formation. Here, we used the mouse dermis as a model tissue to address the necessity of CCL21 anchoring to lymphatic capillary heparan sulfates in the formation of interstitial CCL21 gradients. Surprisingly, the absence of lymphatic endothelial heparan sulfates resulted only in a modest decrease of CCL21 levels at the lymphatic capillaries and did neither affect interstitial CCL21 gradient shape nor dendritic cell migration toward lymphatic capillaries. Thus, heparan sulfates at the level of the lymphatic endothelium are dispensable for the formation of a functional CCL21 gradient.

## Introduction

Chemokines and growth factors form extracellular gradients, which guide migrating cells and morphogenetic processes. Endothelial chemokines regulate immune cell approach and transmigration across the blood and lymphatic endothelium. The evidence for mechanisms regulating the extracellular chemokine cues are emerging (1–3). However, the sparsity of endogenous gradients that can be detected at tissue level has hampered research on gradient formation. CCL21 is the only chemokine, which has been directly shown to form functional gradients in tissues at endogenous levels (2, 4), and thus presents a unique opportunity to study the mechanistic basis of formation and maintenance of chemokine gradients.

CCL21 is secreted by lymphatic endothelial cells (LEC), shows the highest concentration at the plasma- and basement membrane of the lymphatic capillary and forms a gradient decaying into the surrounding interstitium (4–7). CCL21 is essential for the guidance of antigen-presenting CCR7 positive dendritic cells (DC) from peripheral tissues to the lymphatic capillaries and further into the parenchyma of lymph nodes (2, 4, 5, 8, 9).

Most gradient forming proteins exist in a soluble and a glycan anchored pool. The binding to proteoglycans, especially heparan sulfates (HS), has been shown to shape fibroblast growth factor gradients by limited diffusion (10, 11). Accordingly, genetic deletion of the essential enzymes of HS synthesis resulted in defective morphogen gradient formation: deletion of *Ext2* and *Extl3*, and thus absence of HS chain polymerization, lead to enhanced FGF diffusion in zebrafish (12), whereas hypomorphic allele of *Ext1* resulted in elevated range of HS binding Indian hedgehog in mouse embryos (13).

CCL21 interacts *in vitro* with glycans like HSs, chondroitin sulfate B and E and also collagen IV *via* its positively charged carboxy-terminus (14, 15). Accordingly, intact lymphatic endothelial HSs are necessary for CCL21 anchoring to the LEC surface in cell culture and the mesenchymal HSs for anchoring to the interstitium of dermal explants (4, 16, 17). Based on these data we hypothesized that LEC produced HSs are causative for CCL21 sequestration at the CCL21 source, i.e. at the lymphatic capillary, *in vivo* and thus regulate the shape of the interstitial CCL21 gradient by limiting the CCL21 diffusion into the interstitium. If true, deletion of LEC derived HSs might lead to flattening of the interstitial CCL21 gradient shape, longer decay length and thus might impair DC migration to the lymphatic capillaries. To test this hypothesis, we specifically abrogated only lymphatic endothelial HS production by a genetic approach and show, surprisingly, that lymphatic endothelial produced HSs are not essential for the formation of a functional CCL21 gradient.

## Materials and Methods

### Mice

All the mice were on a C57BL/6J background. Wild type mice were purchased from Charles River and mTmG mice from Jackson laboratories. *Ext1^flox^* mice were kindly provided by Yu Yamaguchi and Holger Gerhardt (18), *Prox1CreERT2* mice by Taija Mäkinen (19) and *Ccr7* ^−/−^ mice by Reinhold Förster (20). Male and female mice were bred and maintained according to the local rules (Institutional Review Board approval BMWF-66.018/0005-II/3b/2012). *Prox1CreERT2;Ext1^flox/flox^*, *Ext1^flox/flox^*, *Prox1CreERT2;Ext1^flox/flox^;mTmG and Ext1^flox/flox^;mTmG* mice were topically treated with acetone dissolved 4-OHT (10mg/ml) (Sigma-Aldrich) once a day at P2-5. 4-OHT treated *Prox1CreERT2;Ext1^flox/flox^* and *Prox1CreERT2;Ext1^flox/flox^;mTmG* mice are referred to as “*Ext1^ΔLEC^*” whereas 4-OHT treated *Ext1^flox/flox^* and *Ext1^flox/flox^;mTmG* are referred to as “control”. Ears were collected for further analyses or treated with FITC at the age of 4 to 6 weeks. In each experiment control and *Ext1^ΔLEC^* mice were littermates.

### Sorting and Genotyping of Prox1CreERT2;Ext1flox/flox;mTmG Ear Dermal Cells

Ears of the 4-OHT treated *Prox1CreERT2;Ext1^flox/flox^;mTmG* mice were collected at 4 weeks age. Ears were split into ventral and dorsal halves, fat layer was removed, ears were minced and treated with 1 mg/ml Collagenase A (Sigma-Aldrich) in DMEM supplemented with 1.3 mM CaCl_2_ at +37°C for 1 h. The collagenase A treatment was quenched with 10 mM EDTA at room temperature for 10 min, tissue lysates were stripped through 70 µm cell strainer, cells were collected by centrifugation and subsequently resuspended to FACS buffer (5mM EDTA in PBS). FACS Aria (Becton Dickinson) was used to sort the cells to EGFP+ and tdTomato+ populations. Sorted cells were collected by centrifugation and cell pellets were directly lysed with genotyping sample buffer and used for genotyping of *Ext1^flox^* allele, *Ext1* deleted allele and *Oaz1* (loading control).

### Dendritic Cell Preparation

Mature dendritic cells (DCs) were generated by extracting bone marrow from femur and tibia of 8–12 weeks old wild type or *Ccr7* ^−/−^ mice followed by culture in R10 medium (RPMI1640 containing penicillin-streptomycin, glutamine, 10% fetal calf serum; all from Gibco) supplemented with GM-CSF hybridoma supernatant. Day 8 DCs were activated with LPS (200 ng/ml, Sigma-Aldrich) for 20 h. Activated DCs were labeled with 6.7 µM 5-(and-6-) carboxytetramethyl rhodamine, succimidyl ester (TAMRA, Molecular Probes, Life Technologies) in PBS at room temperature for 15 min. The staining reaction was quenched by the addition of R10, cells were collected by centrifugation and resuspended to the R10 + GM-CSF hybridoma supernatant.

### Ear Sheet Preparation, Interstitial Dendritic Cell Migration Assay and Heparinase II Treatment

**Figure 1:**
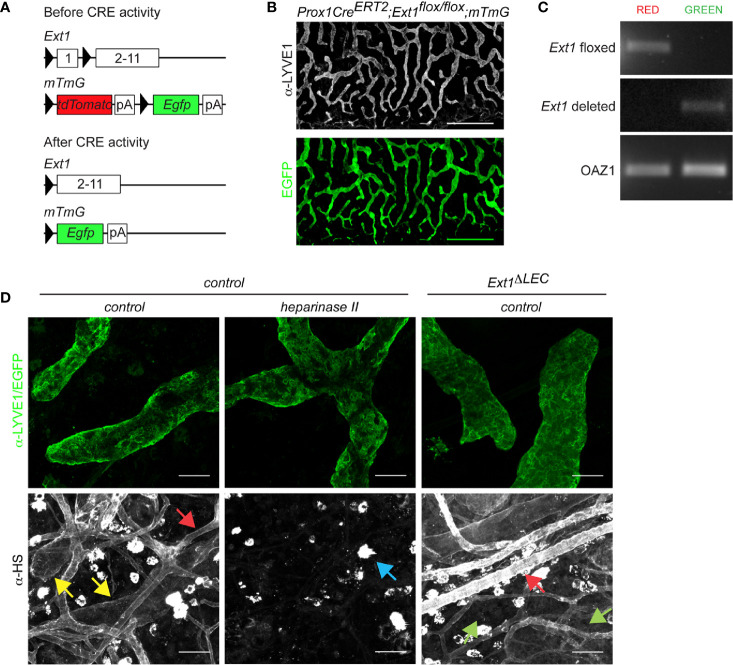
Prox1CreERT2 driven *Ext1* deletion results in a drop of lymphatic endothelial heparan sulfates below detection limit *in vivo*. **(A)** Schematic illustration depicts the used strategy for deletion of Ext1 in lymphatic endothelium. Prox1 promoter mediated expression of CreERT2 and subsequent tamoxifen dependent CRE activation leads to the deletion of the first exon of Ext1 (and thus lack of EXT1 protein production) and concomitant switch- on of the Egfp in LECs. pA stands for polyadenylation signal. **(B)** A wholemount image of mouse ear dermis (EGFP, green; LYVE1 staining, white) of tamoxifen-treated Prox1CreERT2;Ext1^flox/flox^;mTmG mouse (Ext1^ΔLEC^). Scale bar 500 µm. The tiled image was captured with a 10x objective. **(C)** Genotyping of sorted EGFP or tdTomato positive primary cells of two pooled Ext1^ΔLEC^ mouse ears. Image shows PCR product of the Ext1^flox^ allele exclusively in non-recombined red cells and the PCR product of the deleted Ext1 allele exclusively in the recombined green cells. Oaz1 presents a loading control. **(D)** LYVE1 (green) and HS staining (white) of control dermis and HS staining (white) of EGFP expressing Ext1^ΔLEC^ dermis. Heparinase II treatment of control dermis is used as a control for α-HS specificity. Yellow arrows indicate lymphatic and red arrows blood endothelial decoration by HSs. Green arrows indicate the drop of lymphatic endothelial HSs below the detection limit upon Ext1 deletion. Blue arrow indicates heparinase II insensitive bright staining on isolate cells. Scale bar 50 µm. Images were captured with 20x objective.

**Figure 2:**
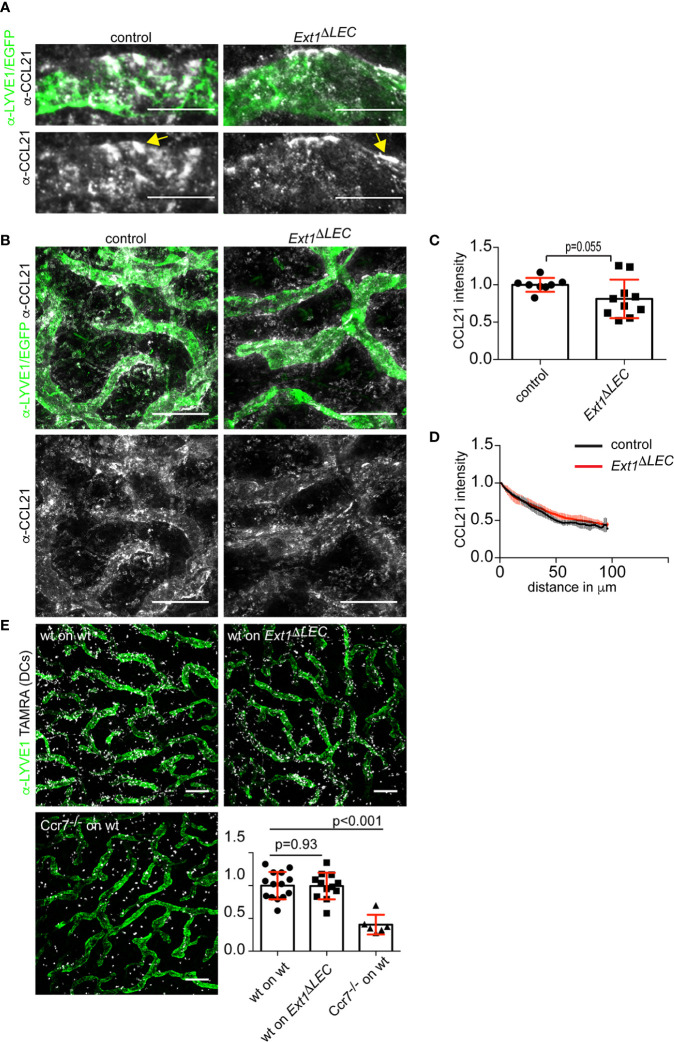
Lymphatic endothelial heparan sulfates are not required for chemokine CCL21 gradient formation. **(A)** High magnification and **(B)** overview images of non-permeabilized CCL21 (white) and LYVE1 (green) stained control dermis or CCL21 (white) stained and EGFP (green) expressing Ext1^ΔLEC^ dermis. Yellow arrow indicates extracellular CCL21 deposits, which possibly represent the sites of DC triggered CCL21 secretion (5). Scale bars 20 and 200 µm, respectively. **(C)** Bar graph shows mean (+/− SD, p-value = 0.055) CCL21 intensity at the lymphatic vessel i.e. CCL21 staining overlapping with LYVE1 staining or EGFP signal (green). N= 8 independent control and Ext1^ΔLEC^ mouse ears. **(D)** Line graph shows a quantification of the mean (+/− SD) interstitial CCL21 intensity in control (black line) and Ext1^ΔLEC^ (red line) mouse ear dermis as a function of distance from the nearest lymphatic vessel margin. N= 4 independent control and five Ext1^ΔLEC^ mouse ears. **(E)** Images show LYVE1 stained (green) lymphatic vessels and TAMRA labeled DCs (white) after 60’ of migration. The associated bar graph shows mean (+/− SD) migration efficiency of DCs toward lymphatic capillaries in control and Ext1^ΔLEC^ ears (p-value = 0.93). The Ccr7^−/−^ DCs are unable to sense CCL21 and thus show random distribution (p-value<0.001). N=14 independent ears for “wt on control” 12 for “wt on Ext1^ΔLEC^” and 6 for “Ccr7^−/−^ on control”. Scale bar 200 µm.

Ear sheets were prepared as reported earlier (4). In short, ears were split and the ventral ear sheets were either fixed with 4% PFA for 25 min (**Figures 1B, D** **)**, placed on PBS for antibody staining of the native ear (**Figures 2A, B** **)** or mounted for interstitial DC migration assay (**Figure 2E**) as follows: ventral ear sheet was placed in between of a 0.5 ml PCR tube lid and the cut top part of the tube, the latter forming a chamber filled with R10. LPS activated TAMRA labeled wild type or *Ccr7* ^−/−^ DCs were loaded on the exposed dermis of control or *Ext1^ΔLEC^* ears and allowed to migrate for 60 min in a cell culture incubator with 5.0% CO^2^ and fixed with 4% PFA at room temperature for 25 min.

As a negative control for heparan sulfate staining (**Figure 1D**), ventral ear sheets were treated with 150 µl of heparinase 2 (3 SU) in PBS supplemented with 0.1% BSA at +37°C for 2 h. Following control or heparinase 2 treatment, ventral ear sheets were washed three times with PBS and fixed with 4% PFA for 25 min at room temperature.

### Ear Sheet Staining

For extracellular CCL21 staining (**Figures 2A, B** **)**, native ventral ear sheets were blocked with 1% BSA in PBS for 45 min. Control *Ext1^flox/flox^;mTmG* ears were incubated with α-LYVE1 and biotinylatedα-CCL21 for 1 h 30 min, washed three times, followed by staining with α-rat Alexa 488 and streptavidin-Alexa 647 for 1 h and washed three times for a total of 30 min with PBS. The *Ext1*^*ΔLEC*^ ears (*Prox1CreERT2;Ext1^flox/flox^;mTmG)* were treated similarly, but the α-LYVE1 and α-rat Alexa 488 were omitted due to lymphatic endothelial EGFP expression (see **Figure 1B**, for the specificity of EGFP expression). Ears were imaged immediately.

For staining of LYVE1 and heparan sulfates (**Figure 1D**) the fixed ears were blocked with 1% BSA in PBS for 1 h. Before antibody staining, 1:50 diluted α-HS (mouse monoclonal IgM, k) (10E4; US Biological) was pre-incubated with biotinylated α-mouse antibody (vector labs) for 60 min at room temperature in blocking buffer. The α-HS (10E4)-anti-mouse antibody complex with or without α-LYVE1 (1:200 dilution; R&D MAB2125) were incubated on blocked ears in blocking buffer for 2 h. The ears were washed three times, incubated with α-rat Alexa-488 secondary antibody and streptavidin-647 and washed three times. All the images were captured with upright Zeiss LSM700 confocal microscopy by using Zen black imaging software.

### Image Analyses

The margins of LYVE1 positive lymphatic vessels (LV) were manually drawn to allow the segmentation of overview images (**Figure 2B**). For the quantification of LV bound CCL21 (**Figure 2C**), the average CCL21 intensity overlapping with the LV mask in control and *Ext1^ΔLEC^* mice was quantified. For CCL21 gradients (**Figure 2D**), CCL21 intensity outside of the mask was measured as a function of distance from the nearest LV mask margin.

For the quantification of interstitial DC migration (**Figure 2E**), DCs were identified by thresholding TAMRA channel images. The distance of each identified DC was measured to the nearest LV margin. As a control of random DC distribution, the LV mask was rotated 90° in relation to the DC image and distances were quantified. The efficiency of migration was evaluated by dividing the real mean distance of DCs to the LVs by the mean distance of the control measurement. Finally, results were presented as normalized to the average of wild type DCs on control ears.

### FITC Painting and Analyses of Lymph Node Cellularity

Ten percent FITC stock solution was dissolved 1:5 in a 1:1 mix of Acetone and DBP (Dibutyl-Phtalate). The 5 weeks old 4-OHT treated control (*Ext1^flox/flox^*) or *Prox1CreERT2;Ext1^flox/flox^* (*Ext1^ΔLEC^*) mice were anesthetized with isoflurane and both the ventral and dorsal sides of the ears were painted with 25 µl of the FITC suspension. Seventy-two hours later, cervical lymph nodes were harvested and placed on ice in RPMI1640 supplemented with 10% FCS and 5 mM EDTA, smashed, centrifuged and resuspended in RPMI1640 supplemented with 10% FCS and 5 mM EDTA. The 3*10^6^ cells/96well were blocked with α-CD16/32 and stained with α-CD11b BV421, α-MHC II (I-A/I-E) BV 510, α-CD103 PE, α-CD8 PerCP-Cy5.5, and α-CD11c PE-Cy7 (BD Biosciences). Subsequent to 45-minute incubation cells were washed once, resuspended in PBS and immediately fixed with 2% PFA at room temperature for 20 min. Fixed cells were washed once, slowly resuspended in PBS 0.25% saponin for 10 min and then stained with α-langerin Alexa-647 (Dendritics) for 30 min. Cells were washed twice, resuspended in PBS and analyzed with FACS Aria.

The stepwise gating strategy was: 1. FSC-A vs. SSC-A gating. 2. FSC-A vs. FSC-W single cells gating. 3. MHCII vs. CD11c gating was used for the identification of a. lymph node resident (MHCII^int^ CD11c^high^) and b. migratory DCs (MHCII^high^ CD11c^int^). 4. Migratory DCs were further defined to 4 populations based on CD103 vs. langerin gating. 5. Upon FITC painting there were no FITC positive cells in the resident DCs population whereas migratory FITC positive cells were abundant. Further, there were no FITC positive cells in the samples derived from non-painted mice.

**Figure 3:**
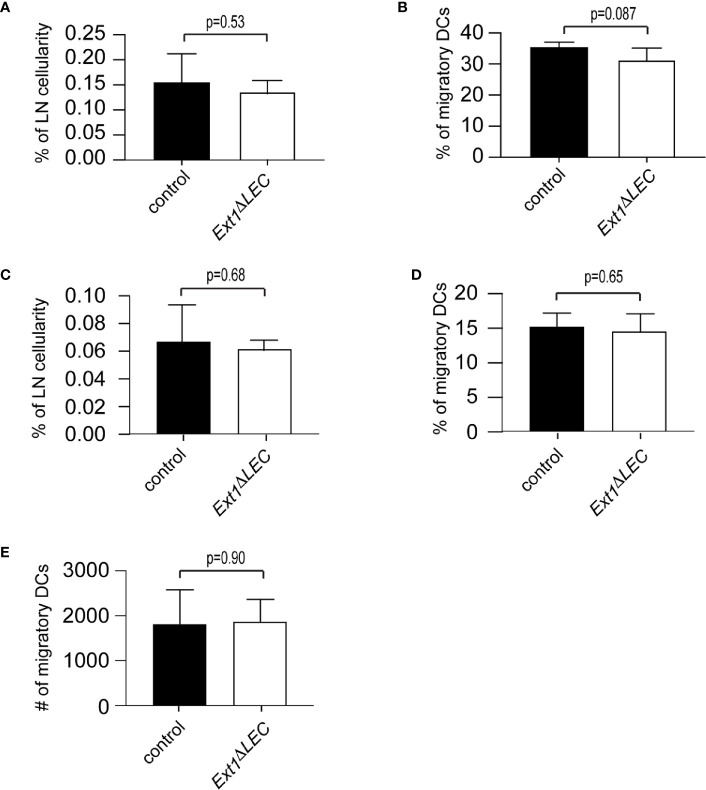
Efficient DC homing to lymph nodes in Ext1^ΔLEC^ mice upon FITC painting. **(A, B)** Bar graphs show mean (+/− SD) percentage of FITC^+^, CD11c^int^, MHCII^high^ DCs of **(A)** total lymph node cellularity (p-value = 0.53) **(B)** and migratory DCs (CD11c^int^, MHCII^high^) (p-value = 0.087) in lymph nodes of control or Ext1^ΔLEC^ mice. **(C, D)** Shows mean (+/- SD) percentage of FITC^+^, CD11c^int^, MHCII^high^, langerin^+^, CD103^-^ Langerhans cells of **(C)** total lymph node cellularity (p-value = 0.68) and **(D)** migratory DCs (CD11c^int^, MHCII^high^) (p-value=0.65) in lymph nodes of control or Ext1^ΔLEC^ mice. **(E)** Shows mean (+/- SD) absolute number of CD11c^int^, MHCII^high^ migratory DCs (p-value = 0.90) in lymph node of control or Ext1^ΔLEC^ mice. N= 5 independent samples/genotype.

The proportion of FITC+ migratory DCs and FITC+ Langerhans cells^langerin+ CD103-^ to all migratory DCs in the cervical lymph nodes and total lymph node cellularity were quantified. The results of left and right cervical lymph nodes from a single mouse were pooled and used as a single data point. The results are shown in **Figure 3**.

### Statistics

Normality of the data was tested with Shapiro-Wilk and Kolmogorov-Smirnov tests and the statistical significance was tested with the Student’s t-test with two-tailed distribution and Welch’s correction (Prism software, GraphPad software).

## Results

To study the contribution of lymphatic endothelial heparan sulfates to CCL21 gradient formation, we specifically prevented HS production in LECs by deletion of *Ext1*, a rate-limiting gene in the HS synthesis pathway. Lack of *Ext1* expression leads to a loss of HSs *in vivo* and *in vitro* (18, 21, 22). To induce the deletion of *Ext1* in the mouse ear dermis lymphatic endothelium, we generated *Prox1CreERT2;Ext1^flox/flox^;mTmG* mice, in which tamoxifen-induced recombination is reported by a switch on of EGFP and switch off of tdTomato (**Figures 1A–C**). Indeed, upon tamoxifen treatment, EGFP was observed exclusively in the lymphatic endothelium (**Figure 1B**), indicating that Prox1CreERT2 has been expressed specifically, and at sufficient levels, in all the LECs of lymphatic capillaries. Genotyping of the extracted mouse ear dermal LECs, marked by EGFP, detected only the deleted *Ext1* allele whereas the non-recombined cell types contained only the non-deleted *Ext1^flox^* allele (**Figure 1C**). Wholemount immunofluorescence staining of mouse ears demonstrated that HSs specifically decorate lymphatic and (more intensely) blood endothelium of control mice. In line with the lymphatic endothelial-specific *Ext1* deletion, HSs were lost below the detection limit specifically in LECs, but not in blood endothelium or interstitium of tamoxifen-treated *Prox1CreERT2;Ext1^flox/flox^;mTmG* mice (**Figures 1C, D**). These results suggest that LECs are the major source of perilymphatic endothelial HS-carrying proteins.

To study the consequence of loss of lymphatic endothelial produced HSs on CCL21 presentation by LECs *in vivo*, we wholemount stained the exposed mouse ear dermis. Extracellular CCL21 levels at the lymphatic capillary of *Ext1^ΔLEC^* mice were only modestly decreased in comparison to control mice (**Figures 2A–C**). Thus, in contrast to earlier *in vitro* results (14, 16, 17), lymphatic endothelium produced HSs are largely dispensable for CCL21 binding to lymphatic capillaries *in vivo*.

Analyses of the interstitial CCL21 intensity showed similar CCL21 gradient shape in control and *Ext1^ΔLEC^* mice (**Figures 2B, D** **)**. Next, we functionally validated these data by *ex vivo* DC migration assays. Activated DCs were loaded onto exposed dermis of split ears, which triggers directed migration of DCs toward the lymphatic capillaries (4). Wild type DCs were as effective in approaching the lymphatic capillaries in control and *Ext1^ΔLEC^* ears whereas *Ccr7* deficient DCs, which are unable to sense CCL21, were randomly distributed in control ears (**Figure 2E**).

To complement our *ex vivo* findings, we compared DC homing to lymph nodes of control and *Ext1^ΔLEC^* mice 72 h after FITC-painting. The proportion of FITC+ migratory DCs of total lymph node cellularity and total lymph node migratory DCs was comparable in control and *Ext1^ΔLEC^* mice (**Figures 3A, B** **)**. Importantly, also the proportion of the FITC+ epidermal subpopulation of DCs, the Langerhans cells, was similar in control and *Ext1^ΔLEC^* mice when compared to the lymph node total cellularity or lymph node migratory DCs (**Figures 3C, D** **)**. Since the peak of the Langerhans cell homing to the draining lymph nodes is at day 4 after immunization (23), this result shows that the first Langerhans cells arrive in lymph nodes efficiently in time also in *Ext1^ΔLEC^* mice, further supporting the existence of normal CCL21 guidance cues in the absence of lymphatic endothelial HSs. Also, the absolute number of lymph node total migratory DCs (FITC+ and FITC−) was unaltered upon deletion of *Ext1* (**Figure 3E**).

## Discussion

Our studies show that, in our model system, LEC produced HSs are dispensable for the formation of the mesenchymal CCL21 gradient (**Figures 2B, D** **)**. We deleted *Ext1* before the establishment of a mature dermal lymphatic vessel network, which resulted in a drop of HSs, both at the LEC surface and basement membrane, below the detection limit (**Figure 1D**). Earlier, it has been shown that binding of CCL21 on the LEC surface *in vitro* is dramatically reduced upon *Ext1* deletion (17). We show that *in vivo Ext1* deletion results only in a modest reduction of the lymphatic capillary associated CCL21 (**Figures 2A–C**). Thus it is conceivable that also *in vivo* HSs are needed for CCL21 binding to the LEC surface, but that the peri-lymphatic capillary matrix, in an HS-independent manner, is the major site of CCL21 anchoring. In support of CCL21 binding on the extracellular matrix at the lymphatic capillary, an earlier study showed that lymphatic capillary associated CCL21 deposits are removed by collagenase treatment (24). The candidate molecules mediating CCL21 anchoring to the cell-matrix in the absence of HS include basement membrane component collagen IV and proteins bearing chondroitin sulfate B and E moieties (14, 15). Interestingly, gradient formation of Indian hedgehog is regulated by both HSs and chondroitin sulfates in mice *in vivo* (13, 25).

We show that in our model system LEC-produced HSs are dispensable for DC migration toward lymphatic capillaries (**Figure 2E**). In contrast, in an earlier study by Bao et al., TEK-Cre-deleter driven *Ext1* deletion in blood and lymphatic endothelium and leukocytes was shown to cause attenuated homing of intradermally injected wild type bone marrow-derived DCs to lymph nodes (17). It is conceivable that blood endothelium or leukocyte HSs are needed for dispersion or sequestration of systemic signals, which affect DC activation and/or migration to lymphatic vessels. In studies by Yin et al., lymphatic endothelial HS production was prevented by deletion of *Ndst1*, an enzyme downstream of EXT1 in the HS synthesis pathway, whose deletion results in altered HS fine structure by preventing N-sulfation. Deletion of lymphatic endothelial *Ndst1* resulted in a decreased xenograft tumor cell metastases to lymph nodes and oxazolone induced lymph node homing of DCs possibly *via* defective CCL21 oligomerization and/or promotion of CCR7-CCL21 interaction rather than anchoring to the matrix (16, 26). It is noteworthy that DC CCL21-CCR7 signaling is dependent on the polysialylation of the receptor (27). Thus, it is conceivable that non-sulfated HSs glycan backbones, created upon *Ndst1* deletion, could interfere with sialic acid-induced conformational changes in CCL21. Our studies also differ in terms of the onset of the prevention of the HS synthesis. Whereas we deleted *Ext1* at early postnatal development to prevent LEC produced HS deposition to the lymphatic capillary basement membrane, Yin et al. deleted *Ndst1* only after maturation of the dermal lymphatic capillary network and deposition of the basement membrane (26, 28).

Recently, Arokiasamy et al. showed a dramatic reduction in HS coverage of lymphatic capillaries upon inflammatory stimulus (29). Interestingly, a decrease in the HS coverage of lymphatic capillaries was essential for efficient tissue fluid drainage, but, importantly, not CCL21 dependent migration and entrance of neutrophils to lymphatic capillaries (29, 30). Together with our study, these results show that CCL21 dependent leukocyte migration cues are not affected by the changes in lymphatic endothelial HSs. However, it is conceivable that the HS may regulate DC trafficking in a CCL21 independent manner *via* mechanisms that were not captured by our model system, for example *via* tissue fluid drainage.

**Figure 4:**
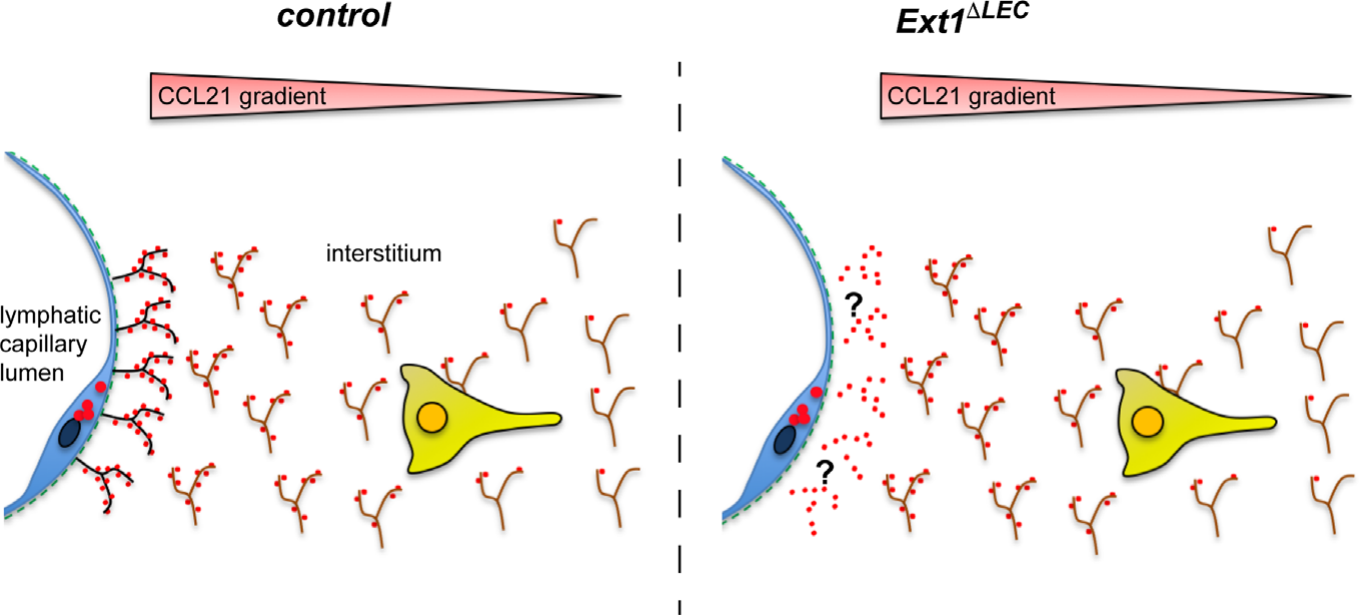
Lymphatic endothelium produced HSs are dispensable for the formation of the interstitial CCL21 gradient. In the absence of lymphatic endothelium (blue) derived HSs (black), there is a modest reduction in the CCL21 (red) levels at the lymphatic capillary (see also **Figures 2A–C**). However, the CCL21 gradient anchored to the mesenchymal HSs (brown) is intact (see **Figures 2B, D**) and allows efficient wild type DC approach (yellow) (see **Figure 2E**) toward the lymphatic capillary.

In conclusion, we show that prevention of the HS production at the source of the CCL21 production does not prevent handing over of the CCL21 chemokine to the mesenchymal HSs (**Figure 4**), which are necessary for the interstitial CCL21 anchoring and gradient (4). To our knowledge, earlier studies showing a necessary role for HSs in growth factor/morphogen gradient formation have deleted HS production in all the cell-types or a clone of all the cells. Here, we have been able to delete HSs only at the source of CCL21 production, leaving HSs at the site of the gradient (interstitium) intact (**Figure 4**). These results highlight the significance of the microenvironment on CCL21 binding and show that not the molecular identity but rather the presence of any diffusion limiting interactions at the lymphatic capillary is sufficient for regulation of CCL21 gradient shape and decay length.

## Data Availability Statement

The raw data supporting the conclusions of this article will be made available by the authors, without undue reservation.

## Ethics Statement

The animal study was reviewed and approved by the Austrian Federal Ministry of Science, Research and Economy (identification code: BMWF-66.018/0005-II/3b/2012).

## Author Contributions

KV and MS conceptualized the study. KV and CM performed the experiments. KV and RH analyzed the data. KV wrote the manuscript with contributions from MS. All authors contributed to the article and approved the submitted version.

## Funding

This work was supported by Sigrid Juselius fellowship (KV), University of Helsinki 3-year research grant (KV), Academy of Finland Research fellow funding (315710, to KV), the European Research Council (ERC CoG 724373 to MS), and by the Austrian Science foundation (FWF) (Y564-B12 START award to MS).

## Conflict of Interest

The authors declare that the research was conducted in the absence of any commercial or financial relationships that could be construed as a potential conflict of interest.
